# Linking the resistome and plasmidome to the microbiome

**DOI:** 10.1038/s41396-019-0446-4

**Published:** 2019-05-30

**Authors:** Thibault Stalder, Maximilian O. Press, Shawn Sullivan, Ivan Liachko, Eva M. Top

**Affiliations:** 1 Department of Biological Sciences, University of Idaho, Moscow, ID 83844 USA; 2 Institute for Bioinformatics and Evolutionary Studies, University of Idaho, Moscow, ID 83844 USA; 3 Phase Genomics Inc, Seattle, WA 98109 USA

**Keywords:** Microbiology, Environmental microbiology, Microbial ecology, Clinical microbiology, Microbial communities

## Abstract

The rapid spread of antibiotic resistance among bacterial pathogens is a serious human health threat. While a range of environments have been identified as reservoirs of antibiotic resistance genes (ARGs), we lack understanding of the origins of these ARGs and their spread from environment to clinic. This is partly due to our inability to identify the natural bacterial hosts of ARGs and the mobile genetic elements that mediate this spread, such as plasmids and integrons. Here we demonstrate that the in vivo proximity-ligation method Hi-C can reconstruct a known plasmid-host association from a wastewater community, and identify the in situ host range of ARGs, plasmids, and integrons by physically linking them to their host chromosomes. Hi-C detected both previously known and novel associations between ARGs, mobile genetic elements and host genomes, thus validating this method. We showed that IncQ plasmids and class 1 integrons had the broadest host range in this wastewater, and identified bacteria belonging to *Moraxellaceae*, *Bacteroides*, and *Prevotella*, and especially *Aeromonadaceae* as the most likely reservoirs of ARGs in this community. A better identification of the natural carriers of ARGs will aid the development of strategies to limit resistance spread to pathogens.

## Introduction

Multi-drug resistant pathogens are increasing in prevalence worldwide [1–3]. The alarming rate at which bacteria adapt to antibiotics is partly due to their ability to acquire antibiotic resistance genes (ARGs) through horizontal transfer of mobile genetic elements (MGEs) such as plasmids. For example, plasmid-mediated resistance has emerged against quinolones [4], carbapenems [5], and colistin [6]. In addition, other genetic elements such as integrons facilitate the acquisition and expression of ARGs by bacteria [7].

Numerous studies have revealed the diversity, abundance, and distribution of ARGs in habitats such as soil, rivers, human and animal guts, and wastewater treatment plants (WWTPs), implicating them all as plausible reservoirs for ARGs [8]. Cultivation-independent metagenomics of environmental samples is a popular approach, but it often cannot assign a specific bacterial host to the ARGs and the MGEs they are encoded on [9]. Specifically, it cannot identify the hosts of plasmids because total DNA extraction disconnects plasmids and chromosomes. This host-plasmid association is nevertheless critical to understand the ecology of antibiotic resistance and the trajectories that bring resistance genes into the clinic [10].

**Fig. 1:**
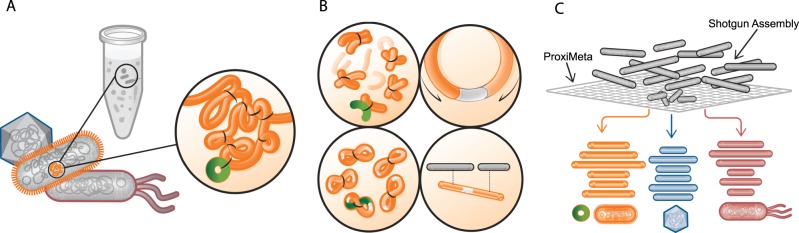
Hi-C deconvolution workflow. **a** Formaldehyde induces covalent bonds between DNAs that are close in three-dimensional space (bonds are depicted by black arcs), and therefore within the same cell. **b** Hi-C library preparation via restriction enzyme digestion (top left), ligation and junction enrichment (bottom left), preparation of the fragment for paired-end sequencing (top right), reads from fragments generated by Hi-C provide linkage information of non-contiguous DNA originated from the same cell (bottom right). **c** Hi-C links can be used in a graph clustering context to deconvolute contigs into their original cellular groupings, including both chromosomes and plasmids

Proximity-ligation methods such as Hi-C and 3C have been used to detect interactions between DNA molecules originating in the same cell within microbial communities (Fig. 1). Both methods are able to reconstruct strain- and species-level genomes from mixed bacterial cultures, and correctly link plasmids and phage to their bacterial hosts [11–13]. These methods can also reconstruct metagenome-assembled genomes (MAGs) from bacterial communities such as those of the mammalian gut communities [14–16]. In this study, we showed that cultivation-independent metagenomic Hi-C data can help determine the reservoirs of ARGs and the plasmids and integrons that carry them in a diverse wastewater community. For example, we were able to determine in our wastewater sample that bacteria belonging to the *Moraxellaceae*, *Bacteroides*, *Prevotella*, and *Aeromonadaceae* are the most likely reservoirs of ARGs. It also led to the reconstruction of several novel MAGs.

## Materials and methods

### Sample collection

The wastewater sample was collected in October 2017 at the Moscow WWTP in Idaho (USA). The facility services ~25,000 people and collects mainly domestic wastewater. The sample was collected at the entrance of the WWTP, and 2 ml subsamples were centrifuged at 12,000 × *g* for 3 min. Pellets were archived at −20 °C until further processing.

### Sample processing

Wastewater pellets were thawed on ice. In half of the aliquots we added a known number of *E. coli* K12::gfp containing plasmid pB10::rfp cells [17], hereafter named EC, such that they represented ~10% of the total bacterial community. Wastewater aliquots spiked with EC are named WWEC and aliquots consisting of the wastewater only are named WW.

The EC strain was grown overnight at 37 °C from a glycerol stock (−70 °C) in LB broth containing nalidixic acid (50 mg.L^−1^), kanamycin (50 mg.L^−1^), and tetracycline (10 mg.L^−1^). The culture was centrifuged for 3 min at 5000 × *g* and pellets were resuspended with the same volume of PBS. We estimated the number of bacteria in the wastewater and in the *E. coli* culture using three methods as described below.

First a qPCR approach was used to target the 16S rRNA gene [18]. Reactions were carried out in a final volume of 10 µL, with 1 µL of pure and 10-fold diluted total genomic DNA from the WW or the bacterial culture, and the PerfeCta® qPCR ToughMix 2× (Quanta BioSciences™, Beverly, MA, USA). The assays were performed in duplicate with an Applied Biosystems StepOnePlus Real-Time PCR System (Applied Biosystems™, Waltham, MA, USA), and a standard plasmid containing the targeted sequence was used to construct a full standard curve in duplicate. The total bacterial cell count in the *E. coli* culture was estimated by dividing the number of 16S rRNA gene copies by the gene copy number in *E. coli* K12 (7). The total bacterial cell count in the water sample was estimated by dividing the number of 16S rRNA gene copies by 4.9, the average 16S rRNA gene copy number among 10,996 bacteria in the rrnDB database (https://rrndb.umms.med.umich.edu/). The WW sample contained an estimated number of 3.5 × 10^8^ bacteria ml^−1^ and the *E. coli* culture 2.7 × 10^9^ ml^−1^. Using the other two methods, flow cytometry and viable plate counts on R2A agar, the estimated bacterial counts were very similar: 3.3 × 10^8^ and 2.7 × 10^7^ per ml, respectively, for WW (only ~10% of bacteria in wastewater can be cultured), and 3.5 × 10^9^ for the *E. coli* culture with both methods.

For the *E. coli* culture, total genomic DNA was extracted from 2 ml of the overnight culture using the GenEluteTM Bacterial Genomic DNA kit (Sigma-Aldrich, St. Louis, MO, USA).

### Library preparation

Shotgun metagenomic libraries were prepared as follows. Total genomic DNA was extracted and isolated from WW and WWEC pellets using the DNeasy® PowerWater® kit (Qiagen, Venlo, Netherlands). PCR-free Illumina libraries for short insert length sequencing with Hiseq were made by the IBEST Genomics Resources Core (Moscow, ID, USA) using TruSeq® DNA PCR-Free library Prep kit (Illumina, San Diego, CA, USA). The Hi-C libraries were prepared from the WW and WWEC pellets using the ProxiMeta™ Hi-C preparation kit (Phase Genomics, Seattle, WA, USA).

### Sequencing

We pooled the Hi-C and shotgun metagenomic libraries, and sequenced both samples using two lanes of HiSeq 4000, 2 × 150 bp paired-end reads at the University of Oregon sequencing core (Eugene, OR, USA). This produced 269,312,499 and 95,284,717 read pairs for the WW shotgun metagenomic and Hi-C libraries, respectively (ratio Hi-C:shotgun = 0.35), and 291,051,995 and 117,388,834 read pairs for the WWEC shotgun metagenomic and Hi-C libraries (ratio HiC:shotgun = 0.40).

### Data processing

Hi-C data analysis was performed using the ProxiMeta workflow as previously described [15]. Some steps are described in more detail below.

#### Processing of the shotgun sequencing data

Shotgun sequencing data were processed using the following steps. Sequencing adapters were removed using BBDuk (BBTools developed by the Joint Genome Institute) with options k = 23, ktrim = r, mink = 12, hdist = 1, minlength = 50, –tpe, –tbo. Low-quality bases will be trimmed with BBDuk and options qtrim = rl, trimq = 10, minlength = 50, chastityfilter = True.

#### Metagenomic assemblies

Shotgun metagenomic assemblies were created de novo using Megahit with default parameters [19]. De novo assemblies were assessed using MetaQuast [20].

#### Processing of the Hi-C reads

Each set of reads was mapped to the metagenomic assemblies. Mapping was done using the Burrows-Wheeler alignment tool BWA-MEM [21, 22]. All reads that were incorrectly paired, unmapped, not uniquely mapped, mapped with a MAPQ score <20, or read pairs mapping to the same contig (which are not informative for deconvolution) were removed from the analysis.

#### Deconvolution of the Hi-C data

Deconvolution of contigs in the de novo assembly was performed using the ProxiMeta platform [15] that is partly based on the previously described method [12]. Briefly, contigs <1000 bp in size, or which contained fewer than two restriction sites for the relevant enzyme were discarded for purposes of clustering. This dataset was normalized by the number of restriction sites on the contigs and contig Hi-C read coverage (which implicitly accounts for length and abundance, among other characteristics). Finally, the contigs were grouped into clusters based on their Hi-C linkages using a proprietary Markov Chain Monte Carlo algorithm.

#### Annotation of genome clusters

Genome clusters were compared with RefSeq genomes using Mash [23] to identify any close database matches for new genome clusters. Genome clusters were further analyzed using the CheckM [24] lineage_wf workflow with the –reduced_tree option to assess genome quality and estimate high-level phylogenetic placements for each cluster based on single-copy marker gene analysis. Some genome clusters were excluded on the basis of promiscuous interactions with other clusters, quantified as their vertex entropy in the Hi-C graph connecting genome clusters, calculated using the R entropy package [25]. We excluded clusters with vertex entropy higher than 3. We assessed abundance of different organisms as the median of the abundance of constituent contigs >20 kb in size, estimated as kallisto “transcripts per million” [26]. Circos plots were generated using Circoletto [27], and genome alignments to references were analyzed using quast v5.0.0 [28].

#### Marker gene detection in the metagenome assemblies

Detection of marker genes was done using BLAT with the options -minIdentity = 90, and hits for which the coverage of the reference sequence was lower than 80% were discarded. When a contig had multiple hits for the same locus, we selected the best hit (best score obtained by multiplying the coverage by the identity). Detection of the ARGs and plasmids were, respectively, done using the MEGARes database [29] and the PlasmidFinder database [30], accessed in April 2018. The MEGARES database was depleted from all genes for which resistance is conferred by SNPs, multi-drug efflux pumps, and regulators. Detection of the class 1, 2, and 3 integron integrase genes was done using the reference sequences AB709942 (*intI1*), FQ482074 (*intI1*delta1), JX566770 (*intI1*R32_N39 aa329337 mutated + 35aa), JX469830 (*intI2*), and EF467661(*intI3*) [31].

#### Linking plasmid, ARG, and integron contigs to genome clusters

We used a simple heuristic to infer linkages between genome clusters and contigs thought to carry ARGs or plasmid sequences.

For each sample, we considered all Hi-C linkages between contigs of interest and any other contig. We then discarded all contig-cluster linkages represented by only one or two reads. We next used the same criteria to infer all linkages between ARG, integron and plasmid contigs. Phylogenetic analysis of the genomes linked to contigs of interest was performed using the CheckM tree workflow, ape v5.1 [32] and phytools v.0.6-44 [33]. We normalized the number of Hi-C contacts (reads) according to abundance of the plasmid/integron/ARG and abundance of the genome cluster. We discarded contacts <0.01 as spurious. We then summarized all normalized contacts across all contigs corresponding to each plasmid, integron, or ARG family (multiple contigs can correspond to the same plasmid, integron, or ARG family).

#### Taxonomic summaries of Hi-C linkages with plasmid/ARG/integron contigs

As an alternative measure of plasmid/ARG/integron—host interactions that did not depend on the accuracy of genome clusters, we ignored ProxiMeta clustering results and considered only the set of contigs to which each contig of interest (i.e., plasmids, ARGs, or integrons) is connected by Hi-C. We used BLAST to search these contigs, considering only the best hit among hits with high confidence (E-value <1e−20). We mapped these hits to NCBI taxonomic identifier (taxids) and species names where possible, otherwise recording “no hit” as the species name. We then filtered out contigs for which the coverage of the alignment was <80%, contigs for which there was “no hit”, and contigs identified as “uncultured” or “*Candidatus*” bacteria. Finally, we counted the number of Hi-C links to each species name, correcting for contig abundance. Where measured abundance was zero, we replaced it with a small nonzero number (0.1 transcript per million (TPMs)).

## Results and discussion

### Proximity-ligation reconstructs a known plasmid-host association from a wastewater sample

We first validated the ability of Hi-C to assemble the genome of a completely sequenced plasmid-bearing bacterium from a wastewater metagenome. To a portion of a wastewater sample, we added ~7 × 10^7^ CFU/mL of *E. coli* K12::gfp containing the multi-drug resistance plasmid pB10::rfp, hereafter named EC, which represented ~10% of the total bacterial community (the raw sample is designated WW, the spiked sample WWEC). For both the inoculated and uninoculated samples we generated short-read metagenome assemblies and used ProxiMeta Hi-C deconvolution [15] to cluster the metagenomic contigs into putative MAGs. This yielded >1000 clusters of contigs for each sample, of which 51 (WW) and 38 (WWEC) were >80% complete bacterial MAGs, as measured by CheckM [24] (Fig. S1, Tables S1 and S2). In this paper, we use the term “cluster” to describe a cohesive group of contigs belonging to a genome of a microorganism. The EC genome was represented by one large cluster (4.2 Mbp) and three small ones (480 Kbp total) with similar high abundance, producing a >97% complete *E. coli* genome (Fig. S2a, Table S3). Furthermore, Hi-C linkage between pB10::rfp and its host was extremely strong relative to other clusters (Fig. S2b), confirming that Hi-C can accurately ascertain plasmid-host relationships within a natural diverse microbial community.

### Cultivation-independent identification of host-ARG associations

**Fig. 2:**
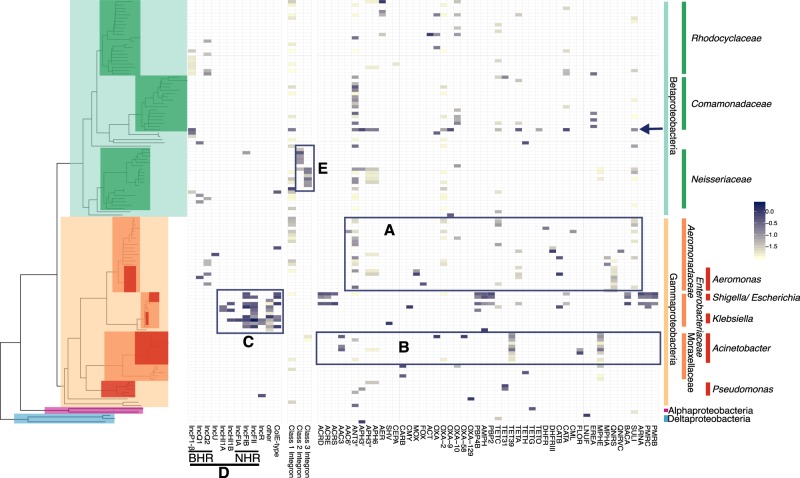
Hi-C links between the clusters and plasmid markers, integrons, and ARGs, identified in the WW sample and affiliated with Alpha- Beta-, Gamma-, and Delta-Proteobacteria. Each tip of the phylogenetic tree represents a cluster. For clarity only clusters having a contact with plasmid markers, integrons, or ARGs are shown (results showing all clusters are presented in Figs. S6 and S7). The presence or absence of a link is shown on the heatmap to the right of the tree, and the color shading represents the intensity of the normalized Hi-C link signals. **a** *Aeromonadaceae* were identified as a natural reservoir of ARGs. **b** Clusters affiliated with the genus *Acinetobacter* showed high Hi-C linkage to ARGs conferring resistance to aminoglycosides, beta-lactams, tetracycline, phenicol, and macrolides. **c** Most plasmids detected belonged to clusters related to *Enterobacteriaceae*. **d** As expected, BHR plasmids were linked to clusters with phylogenetic affiliations broader than the NHR plasmids. **e** Class 2 and 3 integrons were associated with clusters affiliated with the *Neisseriaceae*. The arrow indicates a cluster in *Comamonadaceae* which had a strong link to an IncP-1β plasmid

**Fig. 3:**
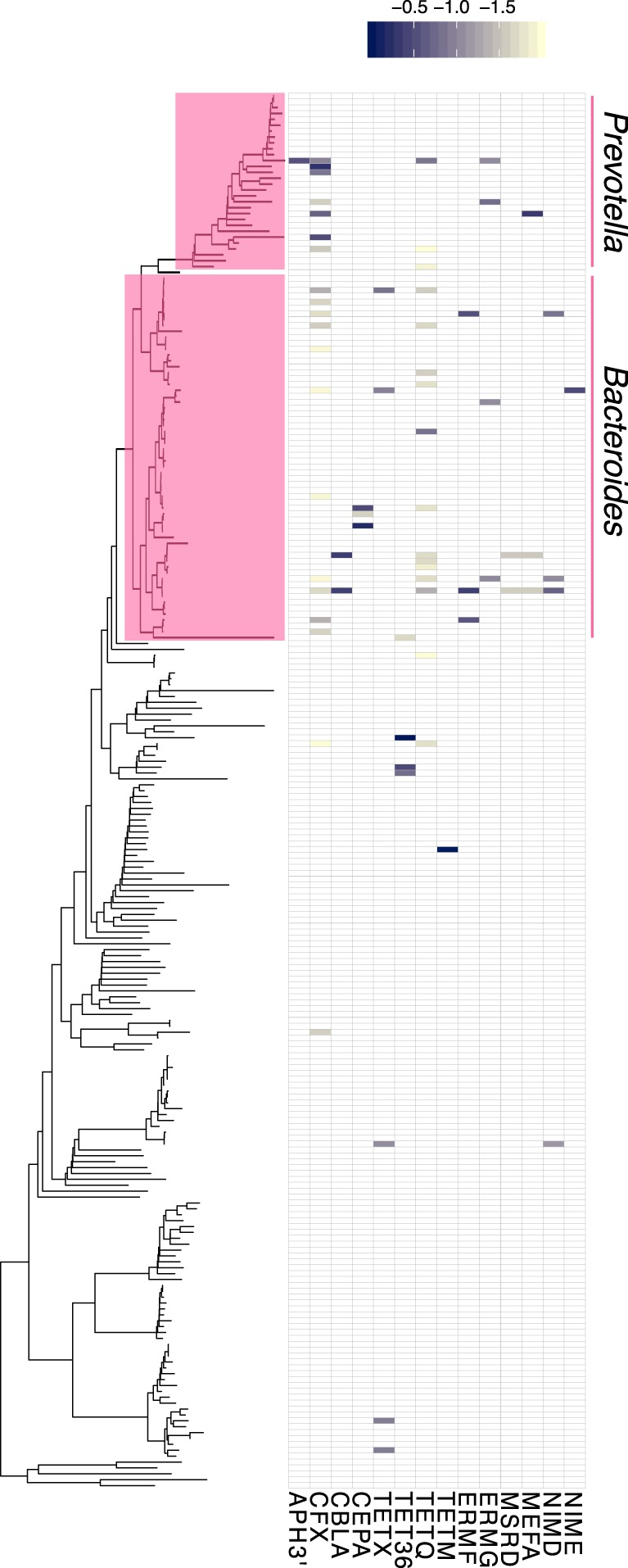
Hi-C linkage between plasmid markers, integrons, and ARGs among clusters belonging to Bacteroidetes in the wastewater sample WW. Each tip of the phylogenetic tree represents a cluster. The presence or absence of a link is shown in the heatmap, with the shading representing the intensity of the normalized Hi-C linkage signal

**Fig. 4:**
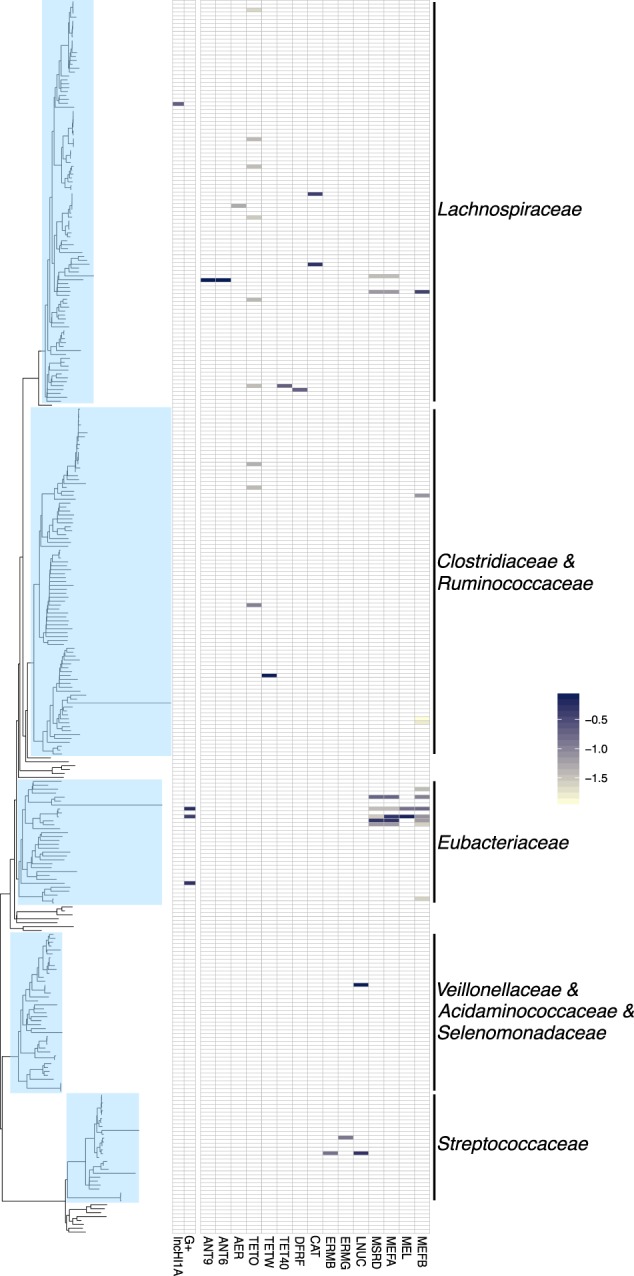
Hi-C linkage between plasmid markers, integrons, and ARGs among clusters belonging to Firmicutes in the wastewater sample WW. Each tip of the phylogenetic tree represents a cluster. The presence or absence of a link is shown in the heatmap, with the shading representing the intensity of the normalized Hi-C linkage signal

We investigated if Hi-C links could be used to identify the hosts in the WW and WWEC samples that contain DNA sequences with high similarity to previously described ARGs, integrons, and plasmids, using well-known databases [29, 30] (Tables S4 and S5). In addition, we inferred phylogenomic placements of each cluster. We then computed the Hi-C linkage of each ARG-bearing or plasmid-bearing contig to each cluster. The results are presented in Figs. 2–4 for WW, and Figs. S3–S5 for WWEC. Most of the analyses described below are results found to be similar in both WW and WWEC, and if not, this is explicitly stated. ARGs were mostly linked to contigs in clusters related to the Gamma- and Betaproteobacteria (Figs. 2 and S3). The other links to ARGs were mostly associated with clusters affiliated with the Bacteroidetes (Figs. 3 and S4) and the Firmicutes (Figs. 4 and S5), and very few with clusters affiliated with Actinobacteria or Fusobacteria (data not shown). Firmicutes showed reproducible linkages to the ARGs *ant9* (linked to *Lachnospiraceae*), *tetO* (linked to *Lachnospiraceae*), *ermB* and *ermG* (linked to *Streptococacceae*), *mefA* and *mefB* (linked to *Proteocatella* sp.), *cat* and *lnuC*. No single Firmicutes family or genus was a predominant host for all of these ARGs but clusters were spread out over the phylum and did not point to a specific reservoir of ARGs.

Reproducible linkages observed in both water samples pointed to specific candidate reservoirs that are well-known carriers of these ARGs. For example, clusters related to *Prevotella* and *Bacteroides*, two genera within the Bacteroidetes abundantly found in gut microbiota, were linked to *tetQ*, *ermG*, *mefA*, *bla*_CFX_, and *bla*_CBLA_, which confer resistance to tetracycline, macrolides/lincosamides/streptogramins (MLS), and beta-lactams, respectively, and are commonly found in these genera (Fig. 3) [34–37]. Specifically *tetQ* and *ermG* or *ermF* are frequently found in tetracycline and erythromycin resistant *Bacteroides* isolates, respectively (*ermF* was only detected in the WW sample) [34, 36]. We also found links with genes encoding the Class A betalactamases Cfx CblA, and CepA (the latter only in WW), which are naturally found in members of *Bacteroides* and *Prevotella* [35, 37, 38].

Similarly, the ARGs *mphE* and *tet39*, recently described on a *Acinetobacter baumannii* plasmid [39], were widespread in several clusters affiliated with the genus *Acinetobacter* (Fig. 2b). Moreover, an *ant3”* resistance gene and a class 3 integron marker were linked with a cluster related an *Acinetobacter johnsonnii*, a species recently described to carry these same genetic elements in a wastewater isolate [40]. In addition, several aminoglycoside resistance genes (*aac3*, *aph3”*, and *aph6*), beta-lactamase genes (*bla*_CARB_ and *bla*_OXA_ variants), tetracycline resistance gene (tetH), and phenicol resistances gene (*floR*) were reproducibly associated with the *Moraxellaceae*.

Interestingly, the bacterial taxa that had the most contacts with known ARGs were affiliated with the *Aeromonadaceae* (Fig. 2a), a family typically associated with aquatic environments. In WW and WWEC, *Aeromonadaceae* were linked to no fewer than 18 and 21 ARGs, respectively (14 being in common), conferring resistance to eight antibiotic classes. Similarly, the contigs linked to clusters of the *Enterobacteriaceae* showed contacts with 18 ARGs in WW and 29 ARGs in WWEC. However, most of these are typically “core structural” genes found in this family, which often only confer reduced susceptibility to antibiotics (i.e., *bla*_EC_, *pmrA, B,C, F, acrD*, *E*, F, *S*, *pbp4b*, *pbp2, ampH*, *arnA*, and *bacA*) [41]. Overall, our results are in line with current knowledge of the taxonomic distribution of the detected ARGs. Hi-C thus suggests that *Aeromonadaceae*, *Moraxellaceae*, and *Bacteroidetes* are the most likely reservoirs of ARGs in this wastewater, consistent with what was suggested in a recent study [42].

### Cultivation-independent identification of host-plasmid associations

Next, we compared the in situ host range of broad-host-range (BHR) and narrow-host-range (NHR) plasmids (Fig. 2c, d). The results were strikingly consistent with our expectations, as two known groups of BHR plasmids (IncQ-1 and IncQ-2) were linked to clusters spanning both *Beta*- and *Gammaproteobacteria*, specifically the *Enterobacteriaceae*, *Aeromonadaceae*, *Neisseriaceae*, *Rhodocyclaceae*, and *Comamomadaceae*. IncP-1β plasmids, also known to be BHR plasmids were widespread in the WW sample but limited to the *Betaproteobacteria*. In WWEC, the marker for IncP1-β plasmids was only associated with the EC cluster, possibly because the links to inoculated IncP1-β plasmid pB10::rfp overwhelmed the analysis (Fig. S3). In contrast, markers for several NHR plasmids were almost exclusively linked to clusters belonging to the *Enterobacteriaceae*. Only one contig with a marker for an IncFIB plasmid was found in a *Betaproteobacteria* cluster, but the link was ~100 times weaker than with the *Enterobacteriaceae*. Another striking finding was the linkage of IncQ and IncU plasmids and a colE-type plasmid with the *Aeromonadaceae*. IncU plamids were originally described in *Aeromonas* sp. [43], and the colE-type plasmid was similar to those described in this genus [44]. Moreover, in WWEC a marker for the IncA/C plasmids was also detected in this family (Fig. S3). IncA/C plasmid markers were also detected in the WW sample but no links to clusters were detected. Just like the IncU plasmids, IncA/C plasmids were first described in an *Aeromonas* species [45]. Further in line with the literature, plasmids previously found in cultured Gram-positives were associated with the Gram-positive family of the *Eubacteriaceae* in this WW (Fig. 4). We show here that the Hi-C method allows assessing the in situ plasmid-host range without any cultivation steps. IncQ plasmids had the broadest range of putative hosts, followed by the IncP-1β plasmids. This is consistent with the results of studies based on cultured bacteria [46].

The method was able to identify the *Aeromonadaceae* as the hosts of the most ARGs as well as several well-known BHR antibiotic resistance plasmids in this wastewater community. This strongly suggests that this family is involved in the spread of antibiotic resistance in WWTP. *Aeromonadaceae* are ubiquitous in aquatic environment, they are frequently isolated from freshwater, estuarine or wastewater, and several species are pathogenic for humans or other vertebrate and invertebrate [47]. We postulate that *Aeromonadaceae* may be an important vector of spread of antibiotic resistance in WWTP. On the other hand, clusters related to the *Enterobacteriaceae* showed links with many plasmids (Fig. 2c), though this may be due to the bias in the plasmid database we used [30]. Although we detected NHR plasmids in this family that are typically involved in antibiotic resistance spread, there were very few links of *Enterobacteriaceae* clusters with ARGs, most of which showed homology to chromosomally and not plasmid encoded genes. This suggests that in this particular community, the *Enterobacteriaceae* were not the main vectors of antibiotic resistance spread.

Finally, in the WW sample, one *Comamonadaceae* cluster with high genome completeness (88.4%; cluster.20) showed strong linkages to an IncP-1β plasmid, a well-known host-plasmid association [48, 49]. We were able to reconstruct two large fragments (22.7 kb and 12.9 kb) carrying the typically conserved transfer regions of IncP1-β plasmids and genes of the maintenance/control region. The closest relative was plasmid pALIDE02 of a WWTP *Comamonadaceae* isolate (see SI, “Intracellular association of ARGs in cluster.20” and Fig. S8).

### Cultivation-independent identification of host-integron associations

Among the integrons, class 1 integrons exhibited links to 39 clusters within the Beta- and Gammaproteobacteria in WW, making it the marker with the broadest host range in this study (Fig. 2). In WWEC no integrons other than the one found in EC were detected, possibly because the strong Hi-C signal of the class 1 integron integrase gene from pB10::rfp carried by EC (Fig S3). Class 2 and 3 integrons were associated with clusters affiliated with the *Neisseriaceae* (Fig. 2e). Whereas class 2 integrons were previously found in a *Neisseria* sp. WWTP isolate (Accession numbers FJ502342 and FJ502343), class 3 integrons were described in a *Delftia* sp. WWTP isolate [50], but so far never in *Neisseriaceae* (Integrall database, http://integrall.bio.ua.pt, consulted on 14 February 2019). This may thus be the first description of class 3 integrons in *Neisseriaceae*.

We conclude that the Hi-C links can help determine the taxonomic placement of the hosts of ARGs and mobile elements in an environmental habitat despite some methodological challenges that will need to be addressed in future work. We describe these challenges below.

### Estimation of the relative abundance of host-marker associations

Like other metagenomics approaches, proximity ligation can only detect associations above some limit of detection imposed by sample complexity and sequencing depth. We attempted to estimate an upper bound on the detection limit of our method for our samples to inform future work applying this technology. This was done by using the abundance of pB10::rfp in WWEC (determined by shotgun read coverage) and the input fraction of its host EC (~10%) to roughly calibrate the relative abundance of hosts linked to other markers (e.g., ARGs or plasmids) (Table S6). Examining several ARGs and plasmid families recapitulating known host relationships, we estimated that these relative host abundances ranged from 0.001% (colE-type plasmids) to 0.5% (*tet39*). While we infer host associations for many elements at the low end of this range, we suggest that a relative host abundance of 0.01% is a conservative estimate of the limit of detection of our method. In other words, we are able to detect host-plasmid associations which are present in one out of 10,000 cells in the sample under the conditions used in this study. This estimate is only an upper bound, and we expect that with further development this detection limit could be lowered. Further validation will require experimental determination of the limit of detection, and also the isolation of specific culturable bacteria to verify their ARGs and plasmid content.

### Limitations of Hi-C

A first limitation we identified is that highly abundant genomes may produce some clustering artifacts. In WWEC, several clusters had Hi-C linkages to chromosomal genes of the EC that was added to the sample (arrows on Figs. S3 and S5). One of these cluster was EC, as expected, but the other clusters were related to the Firmicutes, Alpha- or Betaproteobacteria. The presence of these EC genes in such taxa is very unlikely. We conclude that these Hi-C linkages detected in the WWEC were spurious links probably related to the high abundance of the spiked organism. We also observed Hi-C links between the EC cluster and ARGs or plasmid markers that do not belong to the genome of the spiked strain (i.e., IncFIB, IncFII, and col plasmids, and several ARGs). Such plasmids and ARGs were likely present in other strains of *E. coli* or closely related species present in the wastewater. Indeed, several clusters related to *Escherichia* sp. but different from our strain EC were detected in the WW sample (Fig. 2), some of which had Hi-C links to these genes. Similar observations were made for a cluster that shared some contigs closely related to *E. coli*, and for the cluster.20 described above containing an IncP1-β plasmid very similar to the plasmid pB10::rfp spiked with EC (see details in caption of Figs. S5 and S8, respectively). We therefore conclude that the resolution of contigs that are shared between closely related bacterial species or plasmids may produce spurious links, making it difficult to pinpoint the linkage of a marker to a specific strain. These limitations can be addressed in the future by further optimizing abundance normalization schemes such as the ones we employed in this study, though these require accurate abundance estimates.

### Clustering-independent identification of plasmid and ARG hosts

**Fig. 5:**
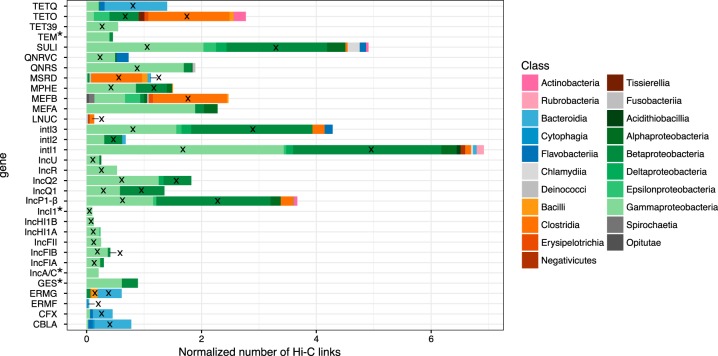
Taxonomic assignment of contigs that were linked to contigs harboring plasmids, ARGs, or integrons. Here the strength of Hi-C linkage is represented by the length of the bars and is summarized by Phylum (pink, Actinobacteria; blue, Bacteroidetes; orange, Firmicutes; green, Proteobacteria, and gray: others), by Class (color shading), and stacked bars of the same color represent different families. *: ARGs or plasmids which did not have links to cluster when using our first approach. The cross indicates that the gene, or marker, was found in the same class when using our first approach

It is possible that ProxiMeta has misclustered contigs, giving us chimeric clusters composed of contigs from different microorganisms. Moreover, the taxonomic assignment done by the lineage-specific marker sets approach used in CheckM may not always have been accurate. We therefore verified the accuracy of our plasmid/integron/ARG host assignments independently of the clusters assembled by ProxiMeta and the taxonomic affiliation inferred by CheckM. We did this by performing taxonomic profiling of all the contigs that were linked to contigs of plasmids, integrons, or ARGs. We first selected these plasmid/integron/ARG containing contigs and then isolated all the contigs linked to these contigs by at least one Hi-C link. Then we used BLAST to find matches of each such linked contig against the NCBI bacterial database (see the “Materials and methods” section). We summarized the host taxonomy of the contigs linked to plasmid/integron/ARG markers in Fig. 5. The new taxonomic affiliation obtained this way were mostly similar to those identified by ProxiMeta (Fig. 2). For example, the MLS resistance gene *mphE* was strongly associated with contigs related to both Gamma- and Betaproteobacteria, more specifically the *Moraxellaceae* and *Neisseriaceae*. Moreover, both quinolone resistance genes *qnrS* and *qnrVC* were mainly associated with contigs of the Gammaproteobacteria, mostly the *Aeromonadaceae*. As in our previous approach, markers for BHR plasmids were linked to clusters spanning the Beta- and Gammaproteobacteria while markers for NHR plasmids were strongly linked to clusters that only belonged mostly to the *Enterobacteriaceae*. Only the phylogenetic attribution of the ARG *mefA* was different between our two approaches.

## Conclusions

While several questions about the accuracy and sensitivity of this approach remain, we show that in vivo Proximity-ligation can help assess the in situ host range of ARGs, plasmids, and integrons in a natural microbial community. We determined for the first-time using cultivation-independent method that IncQ plasmids and class 1 integrons had the broadest host range, confirming these genetic elements as important vectors for horizontal gene transfer. We also concluded that bacteria belonging to the *Aeromonadaceae, Moraxellaceae*, and *Bacteroidetes* are important reservoirs of antibiotic resistance in WWTP, and argued that *Aeromonadaceae* may play a critical role in the spread of antibiotic resistance in WWTP. These analyses can be easily expanded to other MGEs and other habitats. This novel approach fills an important gap in our ability to track the reservoirs and horizontal transfer of antibiotic resistance genes, with the ultimate goal of slowing down the spread of drug resistance.

## Supplementary information

Figure S1

Figure S2

Figure S3

Figure S4

Figure S5

Figure S6

Figure S7

Figure S8

Table S1

Table S2

Table S3

Table S4

Table S5

Table S6

Supplementary information Clean version supplementary information Hi-C ISMEJ

## Data Availability

Sequencing data are available in FASTQ format at SRA accession PRJNA506462. Processed data and scripts for linking contigs to genome clusters using Hi-C data are available at https://osf.io/ezb8j/.
