# Supplementary material for: Linking the resistome and plasmidome to the microbiome
Source: ISME J. 2019 May 30;13(10):2437–46. doi: 10.1038/s41396-019-0446-4 (PMC6776055; doi:10.1038/s41396-019-0446-4)
Supplement: Supplementary file 1 — Figure S1 [file 41396_2019_446_MOESM1_ESM.pdf]

SUPPLEMENTARY INFORMATION

Linking the Resistome and Plasmidome to the Microbiome

Thibault Stalder[1,2], Maximilian O. Press[3], Shawn Sullivan[3], Ivan Liachko[3], Eva M. Top[1,2]

[1]Department of Biological Sciences, University of Idaho, Moscow, Idaho, USA

[2]Institute for Bioinformatics and Evolutionary Studies, University of Idaho, Moscow, Idaho, USA

[3]Phase Genomics Inc., 4000 Mason Rd, Seattle, WA 98195.

Intracellular association of ARGs in cluster.20

For the WW sample, we extracted the subgraph of Hi-C contacts between the 1497 contigs within cluster.20 (including plasmid, integron, and ARG contigs that had Hi-C contacts but were not clustered to cluster.20). We used Louvain clustering of this subgraph to identify contig components of the Hi-C graph that are associated with plasmids, integrons, and ARGs (figure S8). We found that all but one of the 13 plasmid, integron, and ARG contigs in cluster.20 are co-located in one Louvain subcluster of 31 contigs. To further identify contiguous sequences within this subcluster we performed a *de-novo* assembly of the set of contigs using Geneious 8.1.9 (assembler: geneious using preset parameters "high sensitivity"). Seven of the 31 contigs produced a 22.7kb and 12.9kb fragment carrying all typically conserved transfer regions of IncP1-β plasmids (*tra* and *trb* genes) and genes of the maintenance/control region (pink circles on figure S8). Of the 10 ARGs, bla_{OXA9} and *ant*(3'') did not have a direct Hi-C link with a contig harboring a conserved region of IncP1-β plasmids suggesting they were not located on an IncP-1β plasmid. The remaining genes, *aph(3')Ia*, *tetA*, *aph(3'')Ib-aph(6)-Id*, bla_{OXA2}-*ant*(3'')Ia, *tetC*, *sul1* and *catA* had direct Hi-C links with contigs harboring a conserved region of IncP-1β plasmids, suggesting they were plasmid borne.

Surprisingly, one of the plasmid contigs contained the *dsRed* gene and the *catA* resistance gene used to mark the plasmid pB10, resulting in pB10::rfp. This plasmid was present in the EC strain used to spike the WWEC sample but should not be present in the WW sample. Consistent with that, *dsRed* was not detected by qPCR in the total WW gDNA used to make the shotgun library (data not shown). These findings suggest some level of cross-contamination between the WW and WWEC libraries. The *gfp* gene present in the chromosome of the spiked EC strain was not linked to a cluster in our clustering analysis, and we detected very few Hi-C links between clusters related

to *E. coli* and genes found on pB10::rfp. These results show that the cross-contamination was minor. Since the samples WW and WWEC were the same, such cross-contamination does not affect the overall interpretation of our result. However, it affects our ability to determine whether the ARGs linked to cluster.20 were present in the *Comamonadaceae* or belonged to pB10::rfp. Indeed, seven of the nine potential plasmid-borne ARGs in cluster.20 were the ARGs carried by pB10::rfp. Nevertheless, the presence of IncP-1beta plasmids in this cluster is real, as the 22.7kb fragment carrying the *tra* genes of IncP1-β plasmids was identical (>99% coverage >99% identity) to 18 IncP1-β plasmids other than pB10, and the 12.9kb fragment carrying the *trb* genes of IncP-1β plasmids was identical (>99% coverage and >99% identity) to 33 different IncP1-β plasmids, including pB10. The closest relative to both fragments was the plasmid pALIDE02 of *Alicycliphilus denitrificans* BC, a *Comamonadaceae* isolated from WWTP [1]. Members of the *Comamonadaceae* and more broadly the Burkholderiales are well-known hosts of IncP1-β plasmids [2–4]. We concluded that the WW sample contained native IncP1-β plasmids present in *Comamonadaceae* but that many of the ARGs detected may come from the pB10::rfp cross-contamination. This result highlights one of the limitations of the approach when it comes to resolving the location of mobile genetic elements shared among different bacteria (see section "Limitations" in main text).

SUPPLEMENTARY FIGURE LEGENDS

Figure S1: Completeness and contamination of each ProxiMeta genome cluster in each sample, as estimated by CheckM from single-copy marker genes. Current thresholds used to classify the quality of draft genomes recovered from metagenomic assembly, commonly named MAG (Metagenome Assemble Genome), were defined by the authors of CheckM [5]. The completeness of MAG and their associated brackets in percentage were defined to be “near” (≥90%), “substantial” (≥70% to 90%), “moderate” (≥50% to 70%), and “partial” (<50%). The contamination of MAG and their associated brackets in percentage were defined to be “low” (≤5%), “medium” (5% to ≤10%), high (10% to ≤15%), and very high (>15%).

Figure S2: (A) Alignment of the EC cluster to the reference genome of the *Escherichia coli* K-12 MG1655 genome and the plasmid pB10::rfp. Threshold for blast was $E<10^{-100}$. Coloring represents the percent pairwise nucleotide identity with blue ≤ 50%, green ≤ 79.9%, orange ≤ 89%.9, red > 89.9 % identity. The assembled genome cluster EC was composed of cluster.11 (44 contigs, 4.18Mb) and cluster.1069, cluster.925, and cluster.1037 (32 contigs, 478kb). Aligned against the reference genome sequence produced an alignment covering 97.5% of the reference covered with >99.9% identity. (B) Hi-C linkages faithfully recapitulate a known host-genome relationship between the plasmid pB10::rfp and its *E. coli* K-12 host spiked into the WW sample.

Figure S3: Hi-C links between the clusters and plasmid markers, integrons, and ARGs, identified in the WWEC sample and affiliated to Alpha- Beta-, Gamma-, and Delta-Proteobacteria. Each tip of the phylogenetic tree represents a cluster. For clarity purpose only clusters having a contact with plasmid markers, integrons, or ARGs are shown (results showing all clusters are presented in Fig.

S6). The presence or absence of a link is shown on the heatmap to the right of the tree, and the color shading represents the intensity of the normalized Hi-C link signals. **A)** *Aeromonadaceae* were identified as a natural reservoir of ARGs. **B)** Clusters affiliated with the genus *Acinetobacter* showed high Hi-C linkage to ARGs conferring resistance to aminoglycosides, betalactams, tetracycline, phenicol and macrolides. **C)** Most plasmids detected belonged to clusters related to *Enterobacteriaceae*. **D)** As expected, BHR plasmids were linked to clusters with phylogenetic affiliations broader than the NHR plasmids. **E)** Class 2 and 3 integrons were associated with clusters affiliated with the *Neisseriaceae.* Arrows indicates clusters where Hi-C links suggest the presence of several genes naturally present in the chromosome of *E. coli* K12 (EC) added in WWEC (bla_{EC}, *pmrA, B ,C, F, acrD*, *E*, *F*, *S*, *pbp4b*, *pbp2, ampH*, and *arnA*) . The black arrow specifically shows the EC cluster.11.

Figure S4: Hi-C links between the clusters affiliated with the Bacteroides and ARGs in the WWEC sample. Each tip of the phylogenetic tree represents a cluster. The presence or absence of a link is shown on the heatmap to the right of each tree and the color shading represents the intensity of the normalized Hi-C link signal.

Figure S5: Hi-C links within the Firmicutes between the clusters and ARGs, plasmid markers, and integrons in the WWEC sample. Each tip of the phylogenetic tree represents a cluster. The presence or absence of a link is shown on the heatmap to the right of the tree and the color shading represents the intensity of the normalized Hi-C link signal. Arrows indicates clusters where Hi-C

links suggest the presence of several genes naturally present in the chromosome of *E. coli* K12 (EC) added in WWEC (bla_{EC}, *pmrA, B ,C, F, acrD*, *E*, *F*, *S*, *pbp4b*, *pbp2, ampH*, and *arnA*). This cluster (cluster.1046) representing a total size of 46kb was comprised of 20 contigs for which 12 matched to the reference genome of *E. coli* K12 substr. 1655 with > 99% pairwise identity. The eight remaining contigs either matched the reference genome of *E. coli* K12 substr. 1655 with <99% pairwise identity, different strains of *E. coli* (for five contigs), or unknown or very distant bacteria. However here Proximeta did not cluster this small cluster with the rest of the *E. coli* K12 substr. MG1655 and this small cluster was therefore misplaced in the phylogenetic tree. Finally, one contig with a IncHI1A plasmid marker was linked to a *Firmicutes* cluster, an unlikely association as IncHI1A plasmids are thought to be found only in some Proteobacetria [6]; this was not observed in the WWEC sample.

Figure S6: Hi-C linkage between plasmid markers, integrons, and ARGs among clusters belonging to Alpha-, Beta-, Gamma- and Deltaproteobacteria in the wastewater sample WW. Clusters are arranged in the inner circular phylogenetic tree where each tip represents a cluster. The presence or absence of a link is shown in the heatmap circling the tree, with the shading representing the intensity of the normalized Hi-C linkage signal. Data and features are the same as in Fig. 2 except here all the clusters are shown in the tree and for clarity the ARGs were groups by family.

Figure S7: Hi-C linkage between plasmid markers, integrons, and ARGs among clusters belonging to Alpha-, Beta-, Gamma- and Deltaproteobacteria in the wastewater sample WWEC. Clusters are arranged in the inner circular phylogenetic tree where each tip represents a cluster. The presence or absence of a link is shown in the heatmap circling the tree, with the shading representing the intensity of the normalized Hi-C linkage signal. Data and features are the same as in Fig. S3 except here all the clusters are shown in the tree and for clarity the ARGs were groups by family.

Figure S8: Louvain clustering of Hi-C links within cluster.20 used to identify potential adjacent contigs belonging to plasmids, integrons, or ARGs. The results suggest that the cluster.20 contained an IncP1-β plasmid but that most of the ARGs having Hi-C links to this cluster may have come from the plasmid pB10::rfp, also an IncP1-β plasmids, due to minor cross-contamination between the WW and WWEC libraries.

SUPPLEMENTARY TABLES

Table S1: ProxiMeta report for WWEC sample

Table S2: ProxiMeta report for WW sample

Table S3: Quast reports for specific genomes.

Table S4: Contigs from WW sample identified to harbor an ARG, an integron integrase gene, or a plasmid marker.

Table S5: Contigs from WWEC sample identified to harbor an ARG, an integron integrase gene, or a plasmid marker.

Table S6: Limit of detection estimated from several host-ARG or host-plasmid associations.

REFERENCES

1. Oosterkamp MJ, Veuskens T, Plugge CM, Langenhoff AAM, Gerritse J, Berkel WJH van, et al. Genome sequences of *Alicycliphilus denitrificans* strains BC and K601T. *J Bacteriol* 2011; **193**: 5028–5029.
2. Top EM, Springael D, Boon N. Catabolic mobile genetic elements and their potential use in bioaugmentation of polluted soils and waters. *FEMS Microbiol Ecol* 2002; **42**: 199–208.
3. Krol JE, Penrod JT, McCaslin H, Rogers LM, Yano H, Stancik AD, et al. Role of IncP-1 plasmids pWDL7::rfp and pNB8c in chloroaniline catabolism as determined by genomic and functional analyses. *Appl Environ Microbiol* 2011; **78**: 828–838.
4. Norberg P, Bergström M, Jethava V, Dubhashi D, Hermansson M. The IncP-1 plasmid backbone adapts to different host bacterial species and evolves through homologous recombination. *Nat Commun* 2011; **2**: 268.
5. Parks DH, Imelfort M, Skennerton CT, Hugenholtz P, Tyson GW. CheckM: assessing the quality of microbial genomes recovered from isolates, single cells, and metagenomes. *Genome Res* 2015; gr.186072.114.
6. Suzuki H, Yano H, Brown CJ, Top EM. Predicting plasmid promiscuity based on genomic signature. *J Bacteriol* 2010; **192**: 6045–6055.

SUPPLEMENTARY FIGURES
