# Supplementary figures and images for: Linking the resistome and plasmidome to the microbiome
Source: ISME J. 2019 May 30;13(10):2437–46. doi: 10.1038/s41396-019-0446-4 (PMC6776055; doi:10.1038/s41396-019-0446-4)

# FIGURE S1

WW

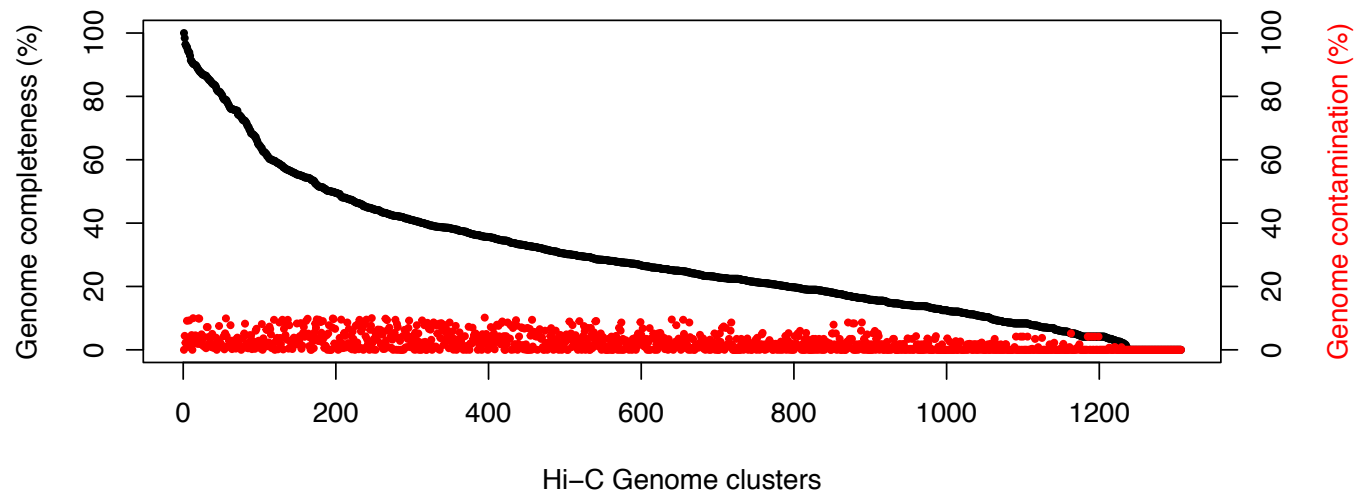

WVEC

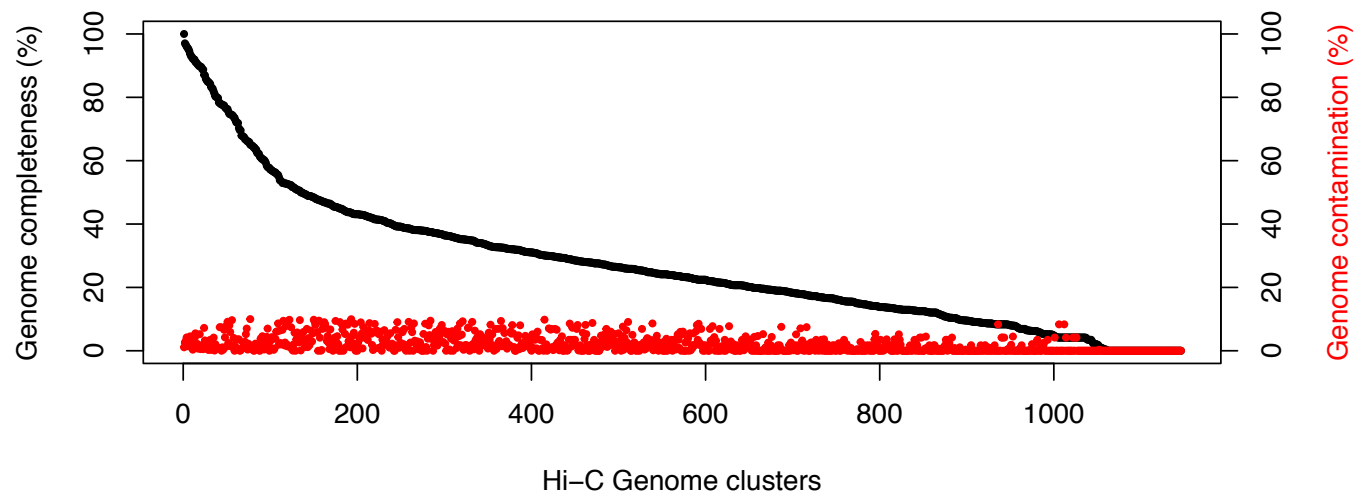

Supplement: Supplementary file 2 — Figure S2 [file 41396_2019_446_MOESM2_ESM.pdf]

FIGURE S2

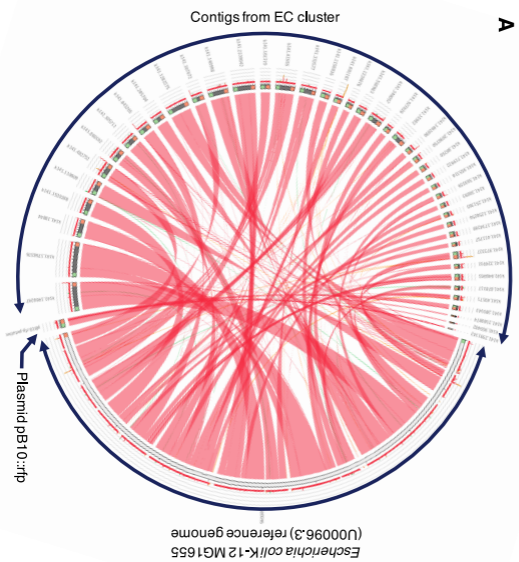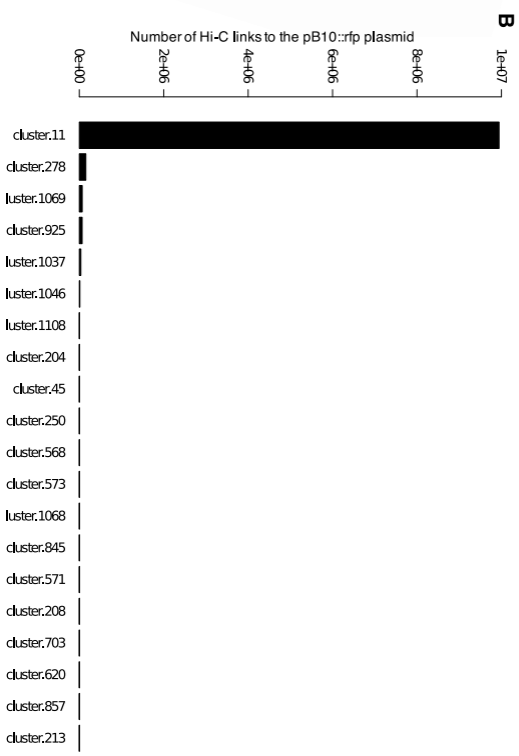

Supplement: Supplementary file 3 — Figure S3 [file 41396_2019_446_MOESM3_ESM.pdf]

FIGURE S3

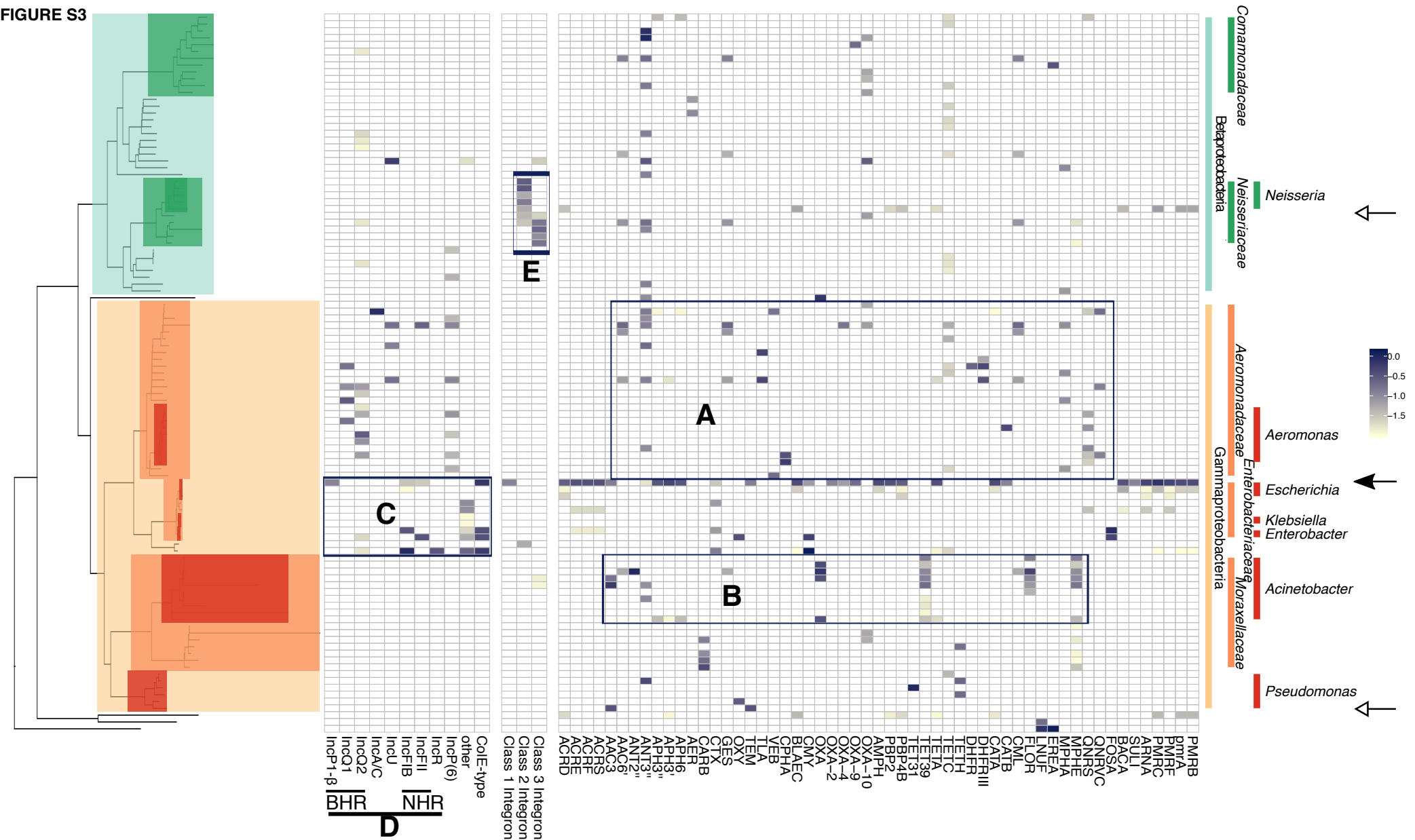

Supplement: Supplementary file 4 — Figure S4 [file 41396_2019_446_MOESM4_ESM.pdf]

FIGURE S4

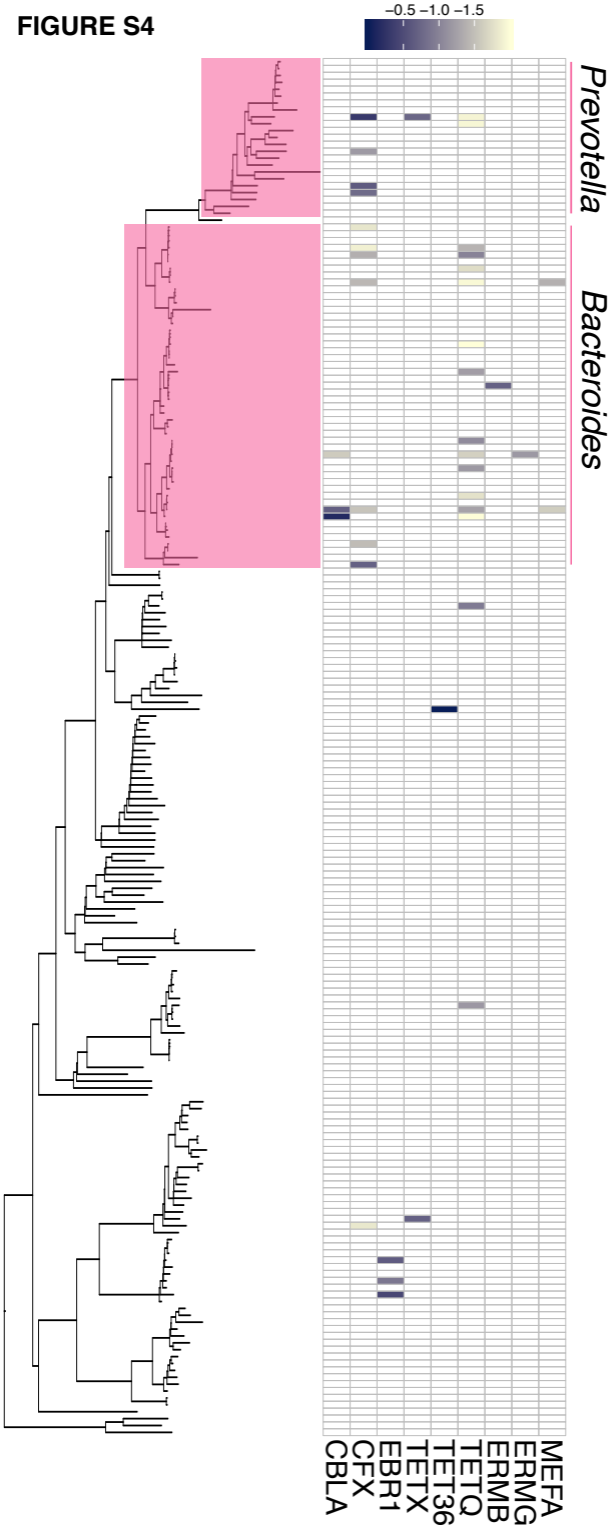

Supplement: Supplementary file 5 — Figure S5 [file 41396_2019_446_MOESM5_ESM.pdf]

FIGURE S5

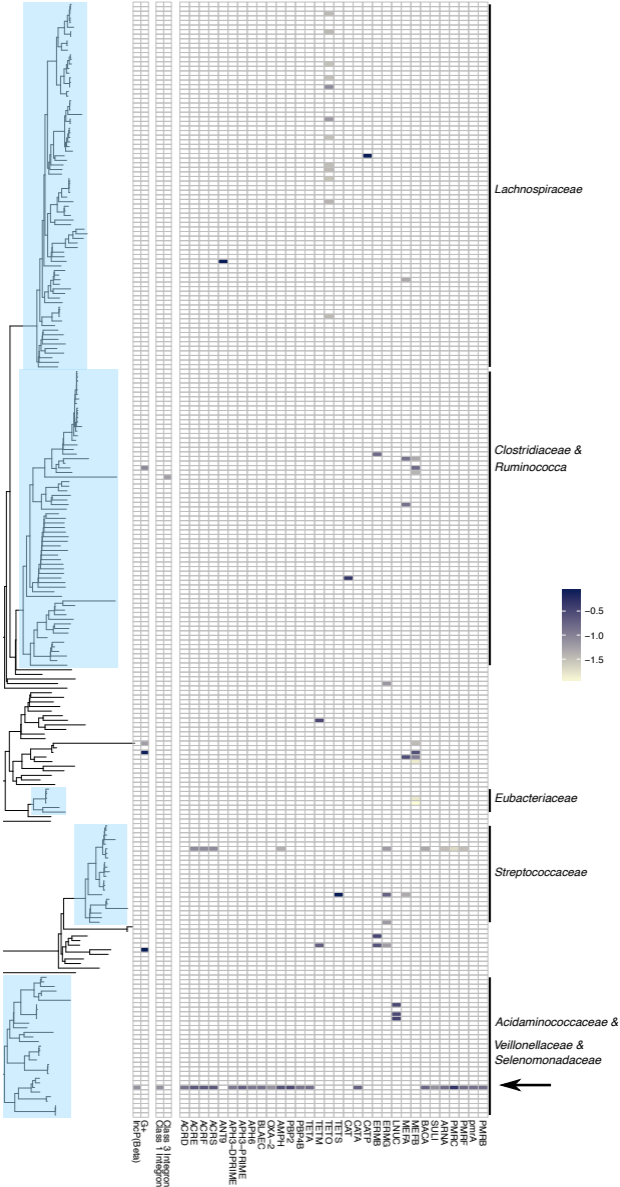

Supplement: Supplementary file 6 — Figure S6 [file 41396_2019_446_MOESM6_ESM.pdf]

FIGURE S6

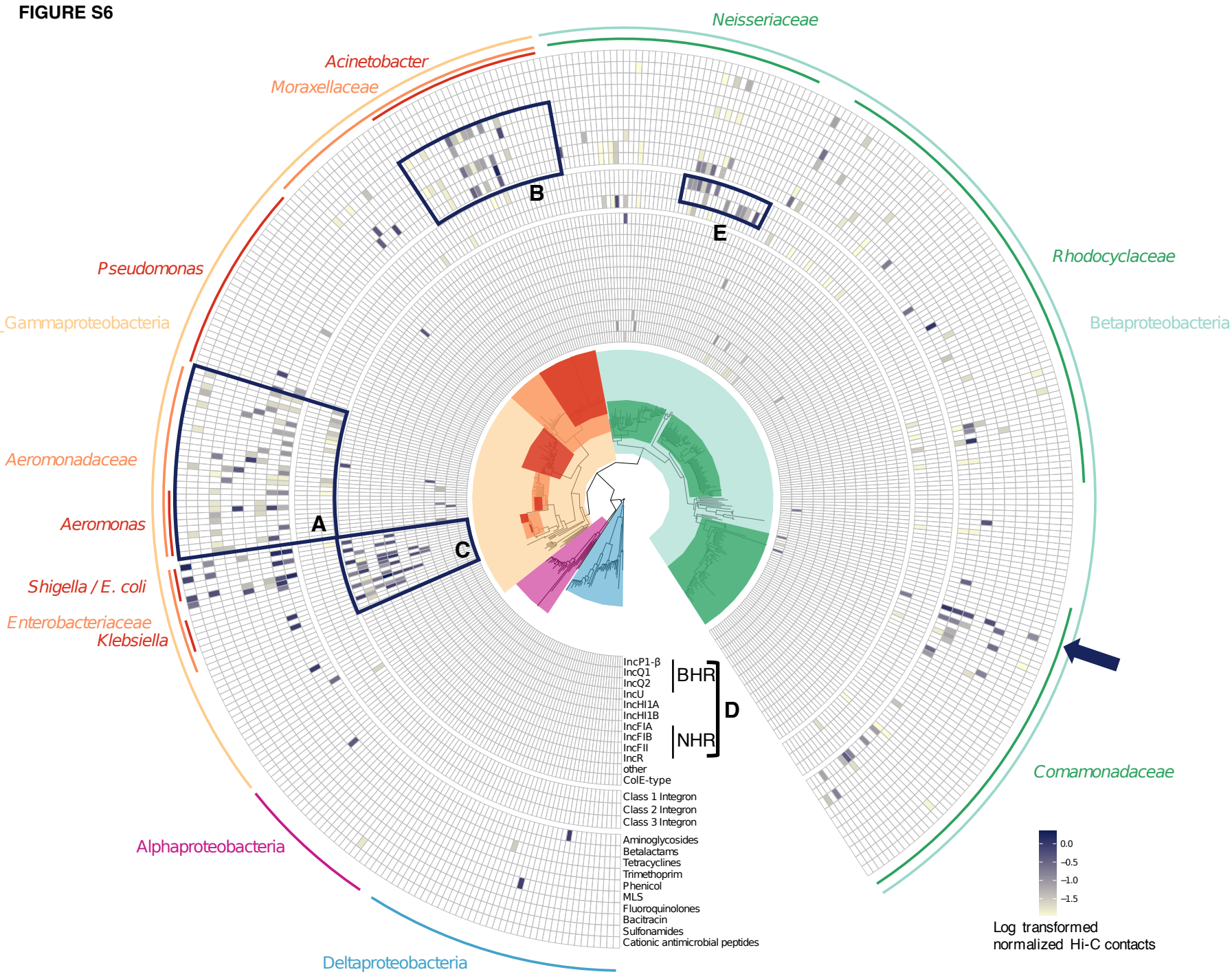

Supplement: Supplementary file 7 — Figure S7 [file 41396_2019_446_MOESM7_ESM.pdf]

**FIGURE S7**

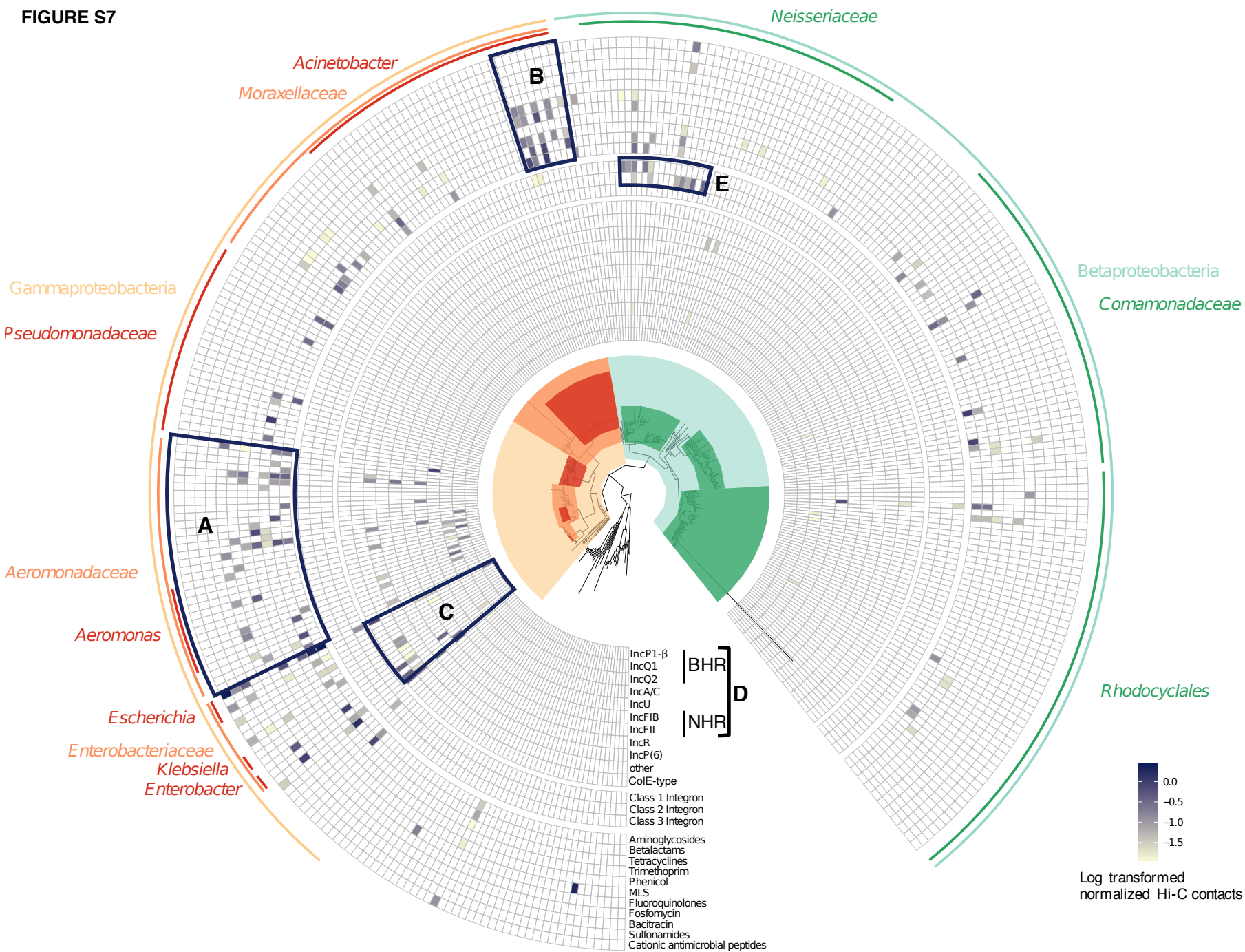

Supplement: Supplementary file 8 — Figure S8 [file 41396_2019_446_MOESM8_ESM.pdf]

# FIGURE S8

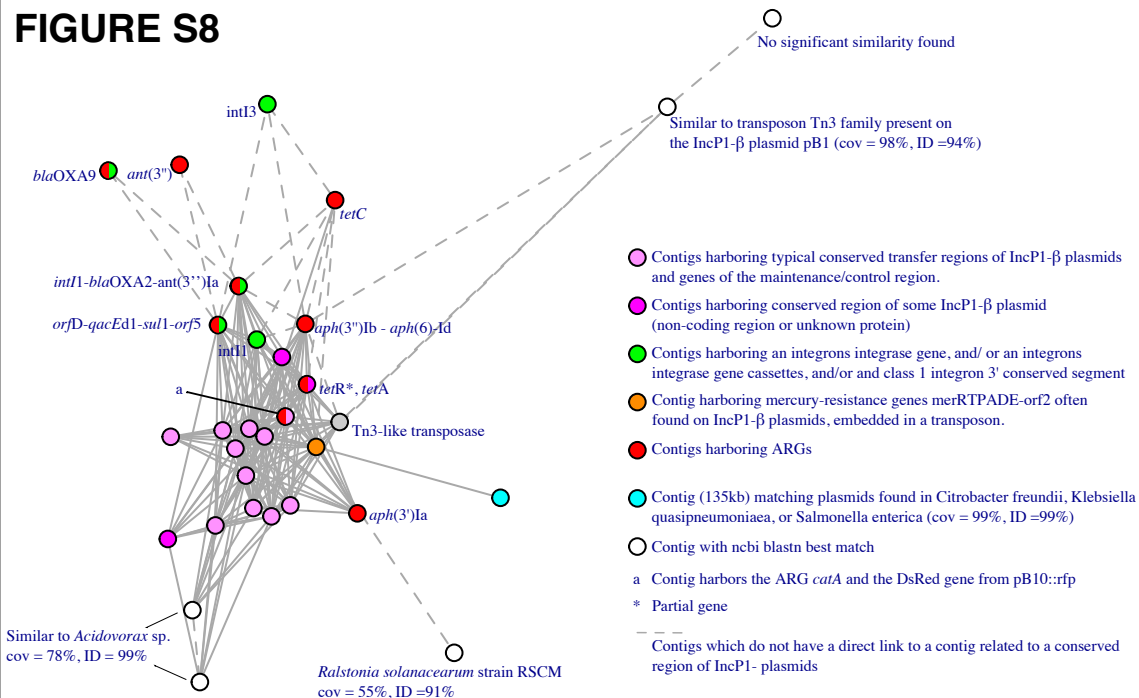

Supplement: Supplementary file 9 — Table S1 [file 41396_2019_446_MOESM9_ESM.pdf]
